# Qualitative and quantitative plaque enhancement on high‐resolution vessel wall imaging predicts symptomatic intracranial atherosclerotic stenosis

**DOI:** 10.1002/brb3.3032

**Published:** 2023-05-01

**Authors:** Li‐Xin Huang, Xiao‐Bing Wu, Yi‐Ao Liu, Xin Guo, Jie‐Shun Ye, Wang‐Qing Cai, Sheng‐Wen Wang, Bin‐ Luo

**Affiliations:** ^1^ Department of Neurosurgery, Sun Yat‐sen Memorial Hospital Sun Yat‐sen University Guangzhou China; ^2^ Department of Neurosurgery, The Eighth Affiliated Hospital Sun Yat‐Sen University Shenzhen China; ^3^ School of Civil Engineering and Transportation South China University of Technology Guangzhou China

**Keywords:** high‐resolution vessel wall imaging, intracranial atherosclerotic stenosis, ischemic stroke, plaque burden, plaque enhancement

## Abstract

**Background and purpose:**

Intracranial atherosclerotic stenosis (ICAS) is a major cause of ischemic stroke (IS), and high‐resolution vessel wall imaging (HR‐VWI) can be used to assess the plaque characteristics of ICAS. This study aimed to qualitatively and quantitatively assess plaque enhancement of ICAS and to investigate the relationship between plaque enhancement, plaque morphological features, and IS.

**Methods:**

Data from adult patients with ICAS from April 2018 to July 2022 were retrospectively collected, and all patients underwent HR‐VWI examination. Plaque enhancement was qualitatively and quantitatively assessed, and the plaque‐to‐pituitary stalk contrast ratio (CR) indicated the degree of plaque enhancement. Plaque characteristics, such as plaque burden and area, were quantitatively measured using HR‐VWI. Furthermore, receiver‐operating characteristic (ROC) analysis was performed to assess the ability of CR to discriminate plaque enhancement. The patients were divided into a symptomatic ICAS group and an asymptomatic ICAS group according to the clinical and imaging characteristics. Univariate and multivariate analyses were performed to investigate which factors were significantly associated with plaque enhancement and symptomatic ICAS. The plaque burden and CR were compared using linear regression.

**Results:**

A total of 91 patients with ICAS were enrolled in this study. ICAS plaque burden was significantly associated with plaque enhancement (*p* = .037), and plaque burden was linearly positively correlated with CR (*R* = 0.357, *p* = .001). ROC analysis showed that the cutoff value of CR for plaque enhancement was 0.56 (specificity of 81.8%). Both plaque enhancement and plaque burden were significantly associated with symptomatic ICAS, and only plaque enhancement was an independent risk factor after multivariate analysis.

**Conclusion:**

Plaque burden was an independent risk factor for plaque enhancement and showed a linear positive correlation with CR. The cutoff value of CR for plaque enhancement was 0.56, and CR ≥ 0.56 was significantly associated with symptomatic ICAS, which was independently associated with plaque enhancement.

## Graphical abstract

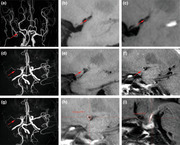

## 1 INTRODUCTION

Ischemic stroke (IS) is the main cause of death and disability worldwide and is a serious threat to human health (Wu et al., 2019; Zonneveld et al., 2018). Intracranial atherosclerotic stenosis (ICAS) is one of the most common causes of IS and is especially common in Asian populations (Holmstedt et al., 2013). ICAS accounted for 46.6% of the acute IS according to the China Intracranial Atherosclerosis (CICAS) Study (Wang et al., 2014). Approximately 40% of recurrent strokes were seen in patients with moderate stenosis (50–69%), as evaluated in the Warfarin versus Aspirin for Symptomatic Intracranial Disease (WASID) trial (Kasner et al., 2006). Recurrent stroke may be strongly associated with the arterial wall of ICAS rather than just being related to the degree of stenosis alone.

With the development of neuroimaging techniques, high‐resolution vessel wall imaging (HR‐VWI) can be used to assess the plaque characteristics of ICAS. Plaque enhancement on HR‐VWI was significantly associated with IS in previous studies, and the recurrence rate of stroke increased with the increasing degree of plaque enhancement (Lee et al., 2018; Song et al., 2021; Sun et al., 2022; Wang et al., 2019). In addition, plaque characteristics, such as plaque burden and plaque area, showed a significant correlation with recurrent acute IS (Ran et al., 2020; Sun et al., 2021). Currently, studies on the quantitative assessment of plaque enhancement and the correlation of plaque characteristics with IS are both lacking. This study aimed to qualitatively and quantitatively assess plaque enhancement of ICAS and to investigate the relationship between plaque enhancement and plaque morphological characteristics, as well as the correlation between plaque characteristics and IS.

## 2 MATERIALS AND METHODS

### 2.1 Study population and data collection

In this study, data from patients with cerebral atherosclerotic stenosis (CAS) who were admitted to our hospital between April 2018 and July 2022 were consecutively collected, and all patients with ICAS identified by CT angiography (CTA), MR angiography (MRA) or digital subtraction angiography (DSA) received HR‐VWI. Clinical data, such as sex, age, height, weight, hypertension, and diabetes, were recorded. The inclusion criteria were as follows: (1) age >18 years; (2) patients diagnosed with ICAS in a large intracranial artery, including intracranial ICA, M1 and M2 segments of MCA, P1 segment of posterior cerebral artery, V4 segment of vertebral artery, and basilar artery, by a prior CTA, MRA, or DSA scan; (3) HR‐VWI of intracranial arteries was performed. The exclusion criteria were as follows: (1) the degree of arterial stenosis higher than 50% in the extracranial segment; (2) other cranial diseases, such as Moyamoya disease, cerebral artery occlusion, cerebral hemorrhage, subarachnoid hemorrhage, and traumatic brain injury; (3) history of atrial fibrillation; (4) history of surgery within 6 months; (5) missing or blurred imaging image data with motion artifact.

### 2.2 Imaging protocol

Three‐dimensional time‐of‐flight MRA, then either 2D or 3D precontrast and contrast‐enhanced T1‐weighted HR‐VWI were scanned on 3T MR scanners. Five min after injection of 0.1 mmol/kg contrast agent, contrast‐enhanced T1‐weighted images were scanned with the same parameters and sites on precontrast T1W imaging. All MR examinations were finished by two well‐experienced technicians. The detailed DSA and HR‐VWI protocols were the same as those in our previous study (Wu et al., 2022), and the parameters were list in the supplementary materials.

### 2.3 Image analysis

#### 2.3.1 Plaque enhancement qualitative and quantitative assessment

Plaque enhancement was defined as the enhancement of the plaque 5 minutes after the intravenous injection of gadolinium and after the signal was highlighted by NMR‐enhanced MRI. Plaque enhancement was assessed independently and in a double‐blinded manner by two experienced readers who were informed only of the site of stenosis and who were blinded to the patient's clinical data and imaging information, and disagreements between the two readers were resolved by consensus.

**FIGURE 1:**
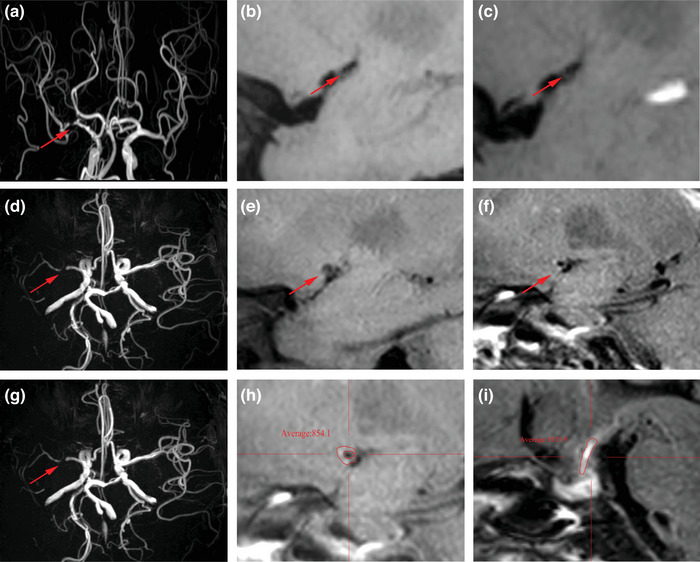
**Case 1**, A 48‐year‐old female with severe stenosis of right middle cerebral artery (R‐MCA). (**a**) R‐MCA stenosis in MRA. (**b**) Precontract HR‐VWI showed the plaque in the R‐MCA. (**c**) The plaque was found not enhancement in postcontract HR‐VWI. **Case 2**, A 54‐year‐old male with severe stenosis of right middle cerebral artery (R‐MCA). (**d**, **g**) R‐MCA stenosis in MRA. (**e**) Precontract HR‐VWI showed the plaque in the R‐MCA. (**f**) The plaque was found enhancement in postcontract HR‐VWI. (**h**, **I**) Mean signal value of R‐MCA plaque and pituitary stalk on postcontrast HR‐VWI.

Using the multiplanar reconstruction tool within the Picture Archiving Communication System (PACS), a T1‐VISTA sequence was selected, and the images were reconstructed in the sagittal or axial plane depending on the orientation of the vessel at the site of the most severe stenosis. The sagittal image was perpendicular to the M1 segment of the middle cerebral artery (MCA), and the axial image was perpendicular to the basilar artery (BA). The mean signal intensity of the plaque (SI_plaque_) and pituitary stalk (SI_stalk_) on the enhanced T1‐VISTA sequence was measured on PACS independently and in a double‐blinded manner by two neurosurgeons. The plaque‐to‐pituitary stalk contrast ratio (CR) was used as the degree of plaque enhancement, CR = SI_plaque_/SI_stalk_, and the mean CR was finally calculated by averaging the CRs (Jiao et al., 2021) (Figure 1). ICC > 0.75 was considered to have good confidence (Bartko, 1966).

#### 2.3.2 Plaque analysis

Plaque area and plaque burden were measured on the maximal stenosis site. Plaque area = vessel area – lumen area (Wu et al., 2022). Plaque burden = 1 – lumen area/vessel area (Wu et al., 2022) (Figure 4 **a and b**). The ICAS lesions were classified as either symptomatic or asymptomatic. A plaque was considered symptomatic if it was the only lesion within the ipsilateral territory of IS or if it was the most stenotic plaque when multiple plaques were present within the same territory of IS. A plaque was defined as asymptomatic if it was not within the vascular distribution of a diffusion‐limited lesion (Yang et al., 2020). Intraplaque hemorrhage (IPH) was defined as the presence of increased T1 SI (>150%) as compared to the adjacent grey matter (Fakih et al., 2020; Saba et al., 2019).

#### 2.3.3 Stenosis analysis

Degree of stenosis = [1 – (*D*_stenosis_/*D*_normal_)] × 100% (Samuels et al., 2000), and the degree of stenosis was divided into different grades: mild (< 50%), moderate (50–69%), and severe (70–99%) (Shi et al., 2020).

#### 2.3.4 Statistical analysis

SPSS 26.0 software (SPSS Company, Chicago, Illinois) was used for statistical analysis. The Shapiro–Wilk test was conducted for continuous variables, the variables consistent with normal distribution were expressed with mean ± standard deviation (SD) and were analyzed comparatively using the *t* test, while the variables does not fit the normal distribution were expressed as medium (min‐max) and were analyzed using Mann‒Whitney *U* test. The categorical variables were analyzed comparatively using the chi‐square test or Fisher test. Variables with *p* < .05 in univariate analysis were included in the multivariate logistic regression analysis to identify the independent risk factors, backward condition was used to calculated the 95% confidence interval (CI) and odds ratio (OR).

Based on our previous study, the ICAS plaques were divided into a plaque enhancement group and a nonenhancement group, and the patients were divided into the symptomatic and asymptomatic groups. The clinical and plaque characteristics of the different groups were compared to clarify which factors were significantly correlated with plaque enhancement and symptomatic ICAS. Then, the independent risk factors for plaque enhancement and symptomatic ICAS were clarified by multivariate analysis. And then factors with *p* < .05 were included in multivariate analysis to clarify the independent risk factors of plaque enhancement and symptomatic ICAS. Moreover, receiver‐operating characteristic curve (ROC) analysis of CR to distinguish plaque enhancement was performed. Linear regression analysis of the linear correlation between plaque burden and CR was performed. In addition, a correlation analysis between CR and plaque burden was performed.

## 3 RESULTS

### 3.1 Clinical characteristics of the patients

A total of 91 patients were recruited in the present study (Figure S1); the mean age was 61.4 ± 9.7 years, and 52 patients (57.1%) were male. Eighty‐two patients underwent a 3D T1 VISTA (motion‐sensitized driven equilibrium, MSDE) sequence scan, while 9 patients underwent a 2D T1BB sequence scan. All patients received DWI inspection (Table 1).

**TABLE 1:** Characteristics of ICAS atherosclerotic plaques with and without enhancement

	Total (*n* = 91)	Enhancement (*n* = 67)	Nonenhancement (*n* = 24)	*p* Value
Age	61.4 ± 9.7	61.8 ± 9.0	59.3 ± 11.6	.279
Sex (male)	52 (57.1%)	42 (62.7%)	10 (41.7%)	.074
BMI	24.8 ± 3.1	24.9 ± 3.1	24.6 ± 3.2	.636
Hypertension	65 (71.4%)	50 (74.6%)	15 (62.5%)	.259
Diabetes	36 (39.6%)	24 (35.8%)	12 (50.0%)	.223
Smoking	27 (29.7%)	21 (31.3%)	8 (33.3%)	.559
Drinking	12 (13.2%)	9 (13.4%)	3 (12.5%)	.908
CHD	9 (9.9%)	7 (10.4%)	2 (8.3%)	.766
Degree of stenosis				.012
70‐99%	58 (63.7%)	48 (71.6%)	10 (41.7%)	
50–69%	17 (18.7%)	8 (11.9%)	9 (37.5%)	
< 50%	16 (17.6%)	11 (16.4%)	5 (20.8%)	
Stenosis site				.475
Anterior circulation	63 (69.2%)	45 (67.2%)	18 (75.0%)	
Posterior circulation	28 (30.8%)	22 (32.8%)	6 (25.0%)	
Plaque burden	0.81 ± 0.11	0.83 ± 0.10	0.77 ± 0.12	.037
Plaque area	17.1 ± 12.8	18.6 ± 13.5	13.0 ± 9.7	.067
IPH	8 (8.8%)	8 (11.9%)	0	.076

ICAS: intracranial atherosclerotic stenosis; BMI: body mass index; CHD: coronary heart disease; IPH: intraplaque hemorrhage.

### 3.2 ICAS plaque enhancement analysis

#### 3.2.1 Qualitative and quantitative analysis

Sixty‐seven patients with ICAS showed plaque enhancement on HR‐VWI, while 24 patients showed no enhancement (*κ* = 0.860). In 52 symptomatic ICAS, 47 were enhancing, while 20 were enhancing in 39 asymptomatic ICAS. The median CR was 0.57 (0.13–1.03) in 82 patients (ICC = 0.942).

#### 3.2.2 Plaque enhancement was independently associated with plaque burden

**FIGURE 2:**
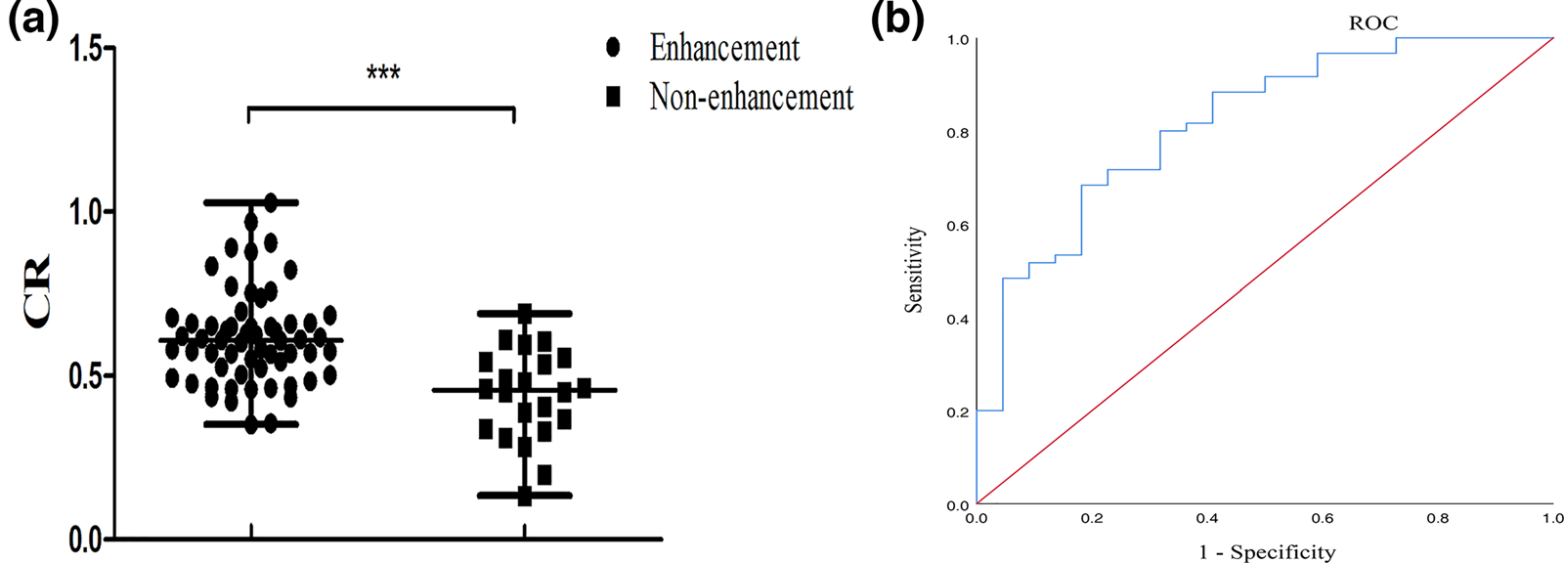
(**a**) The CR of enhancement was higher than that of nonenhancement (0.61 vs. 0.48, ***p < .001). (**b**) ROC curve for determining the presence of plaque enhancement. The cutoff value was 0.56. The AUC of CR was 0.817 (95% CI: 0.714–0.920, *p* < .001). The sensitivity was 68.3%, and the specificity was 81.8%.

A higher degree of stenosis (*p* = .012), greater plaque burden (*p* = .037), and greater CR (0.62 ± 0.14 vs. 0.44 ± 0.14, *p* < .001) were shown in the patients with plaque enhancement compared to those without plaque enhancement (Figure 2 **a**). There were no differences in age, sex, body mass index (BMI), hypertension, diabetes, smoking history, alcohol history, stenosis site, plaque area, and IPH between the plaque enhancement and nonenhancement groups (Table 1). The degree of stenosis and plaque burden were then included in multiple logistic regression and showed that only plaque burden (OR: 90.186, 95% CI, 1.059–7683.937, *p* = .047) (Table 2) was independently associated with plaque enhancement. Compared with plaque nonenhancement, the cutoff value of the CR to differentiate plaque enhancement on the ROC curve was 0.56, with 68.3% sensitivity, 81.8% specificity, 91.1% positive predictive value and 48.4% negative predictive value (*p* < .001), and the area under the curve (AUC) was 0.817 (Figure 2 **b)**.

**TABLE 2:** Multiple logistic regression analysis for ICAS atherosclerotic stenosis with plaque enhancement

Variable	Odds ratio	95% Confidence interval	*p* Value
Plaque burden	90.186	1.059–7683.937	.047
Degree of stenosis	1.525	0.837–2.782	.168

In addition, correlation analysis revealed a linear positive correlation between plaque burden and CR (*R* = 0.357, ICC = 0.942, *p* = .001) (Figure 4 **c**).

#### 3.2.3 Plaque enhancement was independently associated with symptomatic ICAS

**FIGURE 3:**
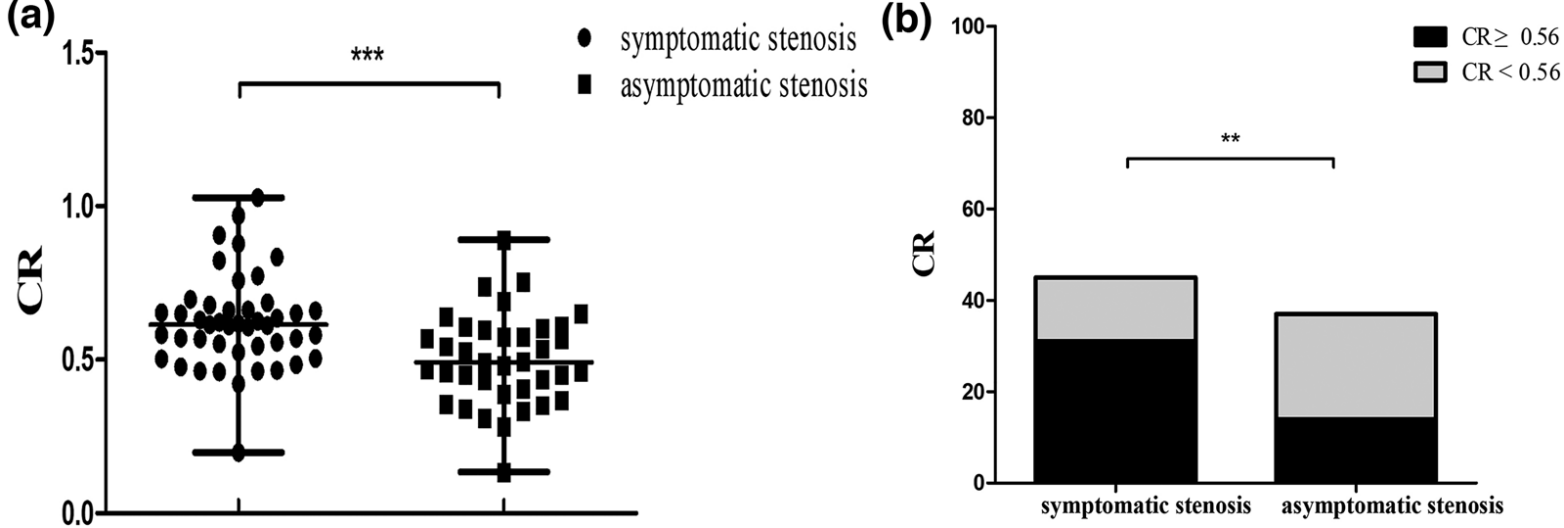
(**a**) The CR of symptomatic ICAS was higher than that of asymptomatic ICAS (0.61 vs. 0.46, ***p < .001). (**b**) There were more symptomatic ICAS in CR ≥ 0.56 group (59.6% vs. 35.9%, **p < .01).

Fifty‐two patients had symptomatic ICAS, and 39 patients had asymptomatic ICAS (*κ* = 0.910). A higher degree of stenosis (*p* = .018), greater CR (*p* < .001), higher proportion of CR ≥ 0.56 (*p* = .005), and a higher proportion of plaque enhancement (*p* < .001) were shown in the symptomatic ICAS group. There were no differences in age, sex, BMI, hypertension, diabetes, smoking history, alcohol history, stenosis site, plaque area, and IPH between the symptomatic ICAS group and the asymptomatic ICAS group (Table 3; Figure 3). Furthermore, stenosis degree, CR, plaque burden and plaque enhancement were included in the multivariate analysis, and the multivariate analysis showed that only plaque enhancement (OR: 8.582, 95% CI, 2.499–29.472, *p* = .001) was independently associated with symptomatic ICAS (Table 4).

**TABLE 3:** Characteristics of ICAS atherosclerotic plaques with and without symptoms

	Total (*n* = 91)	Symptomatic stenosis (*n* = 52)	Asymptomatic stenosis (*n* = 39)	*p* Value
Age	61.1 ± 9.7	61.1 ± 9.9	60.4 ± 9.6	.522
Sex (male)	52 (57.1%)	32 (61.5%)	20 (51.3%)	.328
BMI	24.8 ± 3.1	24.6 ± 3.1	25.1 ± 3.1	.490
Hypertension	65 (71.4%)	41 (78.8%)	24 (61.5%)	.071
Diabetes	36 (39.6%)	25 (48.1%)	11 (28.2%)	.055
Smoking	27 (29.7%)	14 (26.9%)	13 (33.3%)	.508
Drinking	12 (13.2%)	7 (13.5%)	5 (12.8%)	.929
CHD	9 (9.9%)	6 (11.5%)	3 (7.7%)	.543
Degree of stenosis				.018
70‐99%	58 (63.7%)	39 (75.0%)	19 (48.7%)	
50–69%	17 (18.7%)	5 (9.6%)	12 (30.8%)	
< 50%	16 (17.6%)	8 (15.4%)	8 (20.5%)	
Stenosis site				.359
Anterior circulation	63 (69.2%)	38 (73.1%)	25 (64.1%)	
Posterior circulation	28 (30.8%)	14 (26.9%)	14 (35.9%)	
Plaque burden	0.81 ± 0.11	0.84 ± 0.08	0.78 ± 0.12	.009
Plaque area	17.1 ± 12.8	17.7 ± 13.7	16.2 ± 11.6	.582
Plaque enhancement	67 (73.6%)	47 (90.4%)	20 (51.3%)	<.001
IPH	8 (8.8%)	7 (13.5%)	1 (2.6%)	.069
CR	0.57 (0.13–1.03)	0.61 (0.20–1.03)	0.48 (0.13–0.98)	<.001
CR ≥ 0.56	45 (54.9%)	31 (68.9%)	14 (37.8%)	.005

ICAS: intracranial atherosclerotic stenosis; BMI: body mass index; IPH: intraplaque hemorrhage; CR: the plaque‐to‐pituitary stalk contrast ratio.

**FIGURE 4:**
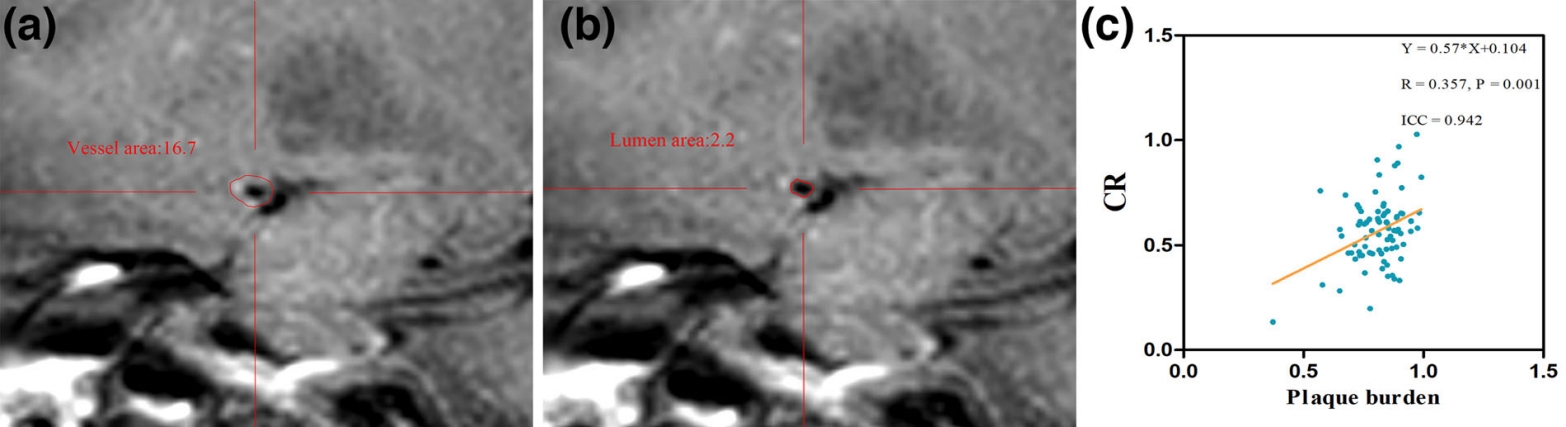
(**a**, **b**) Vessel area and lumen area in HR‐VWI postcontract; (**c**) The linear associations significantly differed between the LMR and CR in 82 test patients. The linear regression equations and correlation coefficient *R* values are provided.

**TABLE 4:** Multiple logistic regression analysis for symptomatic ICAS

Variable	Odds ratio	95% Confidence interval	*p* Value
Plaque enhancement	8.582	2.499–29.472	.001
Degree of stenosis	1.025	0.536–1.962	.940
Plaque burden	136.295	0.772–24077.183	.063
CR	18.734	0.282–1246.135	.171

## 4 DISCUSSION

This study found that the cutoff value for CR to differentiate plaque enhancement was 0.56; furthermore, the proportion of patients with symptomatic ICAS was significantly higher in the CR ≥ 0.56 group than in the CR < 0.56 group. When examining the ICAS plaque characteristics, we found that plaque burden and stenosis were significantly associated with plaque enhancement, and plaque burden was an independent risk factor for plaque enhancement. Moreover, plaque burden showed a linear positive correlation with plaque enhancement. In addition, both plaque burden and plaque enhancement were significantly associated with symptomatic ICAS, and plaque enhancement was an independent risk factor for symptomatic ICAS.

In recent years, HR‐VWI has been increasingly applied to the study of ICAS plaques, and plaque enhancement on HR‐VWI can predict plaque instability. Currently, there are few studies that have performed a quantitative analysis of plaque characteristics. A few studies have recently performed a quantitative analysis of ICAS plaque enhancement and found that CR can help identify culprit plaques (Fakih et al., 2020) and that the plaque enhancement rate (ER) was independently associated with recurrent ischemic stroke (Ran et al., 2020; Sun et al., 2021). In addition, a previous study found that elevated ER was significantly associated with restenosis after stenting (Tian et al., 2022). Fakih et al. (2020) also determined that culprit plaques had significantly higher CR than nonculprit plaques, and CR ≥ 0.53 could differentiate culprit plaques with a specificity of 90%. In this study, CR ≥ 0.56 had a specificity of 81.8% for diagnosing ICAS plaque enhancement; the specificity of our study was lower than that in the previous study, maybe due to the basal CR value of the study was somewhat lower. In addition, CR ≥ 0.56 was significantly associated with symptomatic ICAS. The distinction between enhancement and nonenhancement is sometimes difficult, while quantitative analysis of plaque enhancement is more reproducible. This study suggested that CR ≥ 0.56 should be considered plaque enhancement. CR ≥ 0.56 was significantly associated with symptomatic ICAS, and whether it can predict plaque instability of ICAS needs to be verified in follow‐up studies with large sample sizes.

This study indicated that plaque burden was independently associated with plaque enhancement and that plaque burden had a linear positive relationship with CR. No previous study has found a linear relationship between plaque burden and plaque enhancement. Previous studies have shown that plaque enhancement is a local inflammatory manifestation of plaques (Hur et al., 2010; Millon et al., 2012). In an animal study, Hur et al. (2010) found that plaques with a high degree of enhancement had more macrophage infiltration than plaques with a low degree of enhancement, indicating that when there is a higher rate of enhancement, the plaque has a more pronounced inflammatory response. In addition, Millon et al. (2012) combined HR‐VWI and immunohistochemical techniques in 69 patients with carotid stenosis plaques and found a significant increase in macrophages and neovascularization in the plaque enhancement group, which indicated that plaque enhancement was an inflammatory process. Hellberg et al. (2017) found a significant increase in both plaque burden and macrophages in aortic plaques of mice on a high‐fat diet. Therefore, the present study suggested that the possible reason for the linear positive correlation between plaque burden and CR was that the increased plaque burden, increased local inflammatory cell infiltration, and increased inflammatory response resulted in increased plaque enhancement.

Previous studies have shown a significant correlation between ICAS plaque enhancement and IS, and plaque enhancement is considered an imaging marker of IS (Fakih et al., 2020; Huang et al., 2019; Shi et al., 2020; Wang et al., 2019; Yang et al., 2020). Huang et al. (2019) divided ICAS plaques into culprit and nonculprit groups and confirmed that plaque enhancement was an independent predictor of ischemic events. A longitudinal study found that plaque enhancement was an independent risk factor for IS due to ICAS and that plaque enhancement was the strongest risk factor for plaque progression (Lee et al., 2022; Zhai et al., 2022). In addition, increased plaque enhancement was associated with a significant increase in ICAS progression and recurrent stroke during follow‐up (Hellberg et al., 2017; Zhang et al., 2021). Plaque burden was also significantly associated with IS (Karlöf et al., 2021; Ran et al., 2020; Shi et al., 2021). High plaque burden in the MCA was directly associated with a high recurrence rate of IS (Ran et al., 2020), and Shi et al. (2021) found that plaque burden, as measured using HR‐VWI, was an independent predictor of recurrent acute IS. In addition, Sanchez et al. (2022) created a multivariate model, concluding plaque gadolinium (Gd) uptake and plaque burden, and showed that plaque enhancement analysis was more accurate for identifying symptomatic plaques. In this study, both plaque enhancement and plaque burden were found to be significantly associated with symptomatic ICAS, and this result was similar to previous studies. Multivariate analysis indicated that plaque enhancement was an independent risk factor for symptomatic ICAS.

In addition, this study found a higher incidence of ICAS with severe stenosis in the plaque enhancement and symptomatic ICAS groups than in the nonenhancement and asymptomatic groups, which was consistent with previous findings, revealing that the risk of acute IS increased with the increasing stenosis degree of ICAS (Makowski & Botnar, 2013; Samuels et al., 2000). Atherosclerotic plaques that protrude into the lumen can block cerebral blood flow and lead to hypoperfusion in the distal area, whereby the decreased perfusion may limit embolic clearance and increase the incidence of IS. The rate of IPH in symptomatic ICAS (13.5%) was higher than that in the asymptomatic (2.6%), the difference did not reach statistical significance maybe due to the relative small sample size of the current study. The result showed that there was correlation between IPH with cerebrovascular event, which was similar with the previous study (Saba et al., 2019).

Several limitations should be considered in this study. First, this was a single‐center retrospective study, due to hospitalized patients with ICAS usually having relatively severe stenosis; the sample size was relatively small in this study, which may lead to selection bias for patients. Second, this was a cross‐sectional study that did not perform a clinical follow‐up to investigate whether CR ≥ 0.56 predicted plaque instability of ICAS. Third, the 2D T1BB sequence and 3D T1 VISTA sequence were used for the patients. Although the MSDE technique was applied to improve the capacity of blood suppression, the two different techniques may influence the assessment of plaque enhancement. However, two independent raters had an excellent agreement in the assessment of plaque enhancement, so we believe that the influence was limited. Finally, we were unable to combine plaque histopathology and neuroimaging for further study due to the difficulty in obtaining the plaque tissues of ICAS.

## 5 CONCLUSION

The cutoff value of CR for plaque enhancement was 0.56, which was significantly associated with symptomatic ICAS. Plaque burden was independently associated with plaque enhancement and showed a linear positive correlation with CR. Plaque enhancement and plaque burden were both significantly associated with symptomatic ICAS, but only plaque enhancement was an independent risk factor for symptomatic ICAS.

## AUTHOR CONTRIBUTIONS

Bin Luo and Sheng‐Wen Wang contributed to the study design. Li‐Xin Huang and Xiao‐Bing Wu wrote the main manuscript text and prepared figures. Jie‐Shun Ye did statistical verification of data. All authors participated in the interpretation and collection of the data. All authors reviewed the manuscript.

## CONFLICT OF INTEREST STATEMENT

The authors declare that they have no competing interests.

### ETHICS STATEMENT

All procedures involved in the clinical study were approved by the Sun Yat‐sen Memorial Hospital Research Ethics Board.

### CONSENT FOR PUBLICATION

All authors agree to publish.

### PEER REVIEW

The peer review history for this article is available at https://publons.com/publon/10.1002/brb3.3032.

## Supporting information


**Supplementary Figure S1**. Flow chart of the selected population.

## Data Availability

The data supporting the funding of this study are available from the corresponding author upon request.
